# Cardiac Amyloidosis in the Setting of a Sarcomatous Pericardial Mass

**DOI:** 10.7759/cureus.40807

**Published:** 2023-06-22

**Authors:** Khaled Deeb, Anatoliy Korzhuk

**Affiliations:** 1 Internal Medicine, West Palm Beach VA Medical Center, West Palm Beach, USA

**Keywords:** heart failure, cardiomyopathy, pericardial mass, pericardial sarcoma, transthyretin amyloidosis, cardiac amyloidosis

## Abstract

Cardiac amyloidosis is a significantly underdiagnosed disease but should be suspected in anyone with restrictive heart physiology. Here, we present a case of a sarcomatous pericardial mass confounding the patient’s progressive diastolic heart failure. Amyloidosis was eventually discovered by piecing together serial transthoracic echocardiogram, functional MRI, and technetium-99m (99mTc) pyrophosphate scintigraphy findings along with a negative lab workup. The presence of the sarcomatous pericardial mass raised the question of whether it played a role in the onset and progression of amyloidosis, but nonetheless, the presence of both diseases rendered multifaceted challenges regarding our patient’s care. Anyone suspected to have amyloidosis should receive appropriate testing for a definitive diagnosis to catch the disease process and offer early treatment, as exciting research is emerging showing transthyretin stabilizers to have a reduction in all-cause mortality.

## Introduction

Amyloidosis is a spectrum of diseases referring to the deposition of abnormal proteins in tissues. Classifications include primary light chain (AL), secondary senile amyloid A (AA), transthyretin (TTR) amyloidosis, dialysis-related, and systemic amyloidosis of poorly understood etiology [1]. TTR is a protein created mainly in the liver that is used to transport thyroxine and retinol. Amyloid TTR (ATTR) is the disrupted quaternary structure of transthyretin, and the fibers aggregate into insoluble fibers that damage organs through deposition [1]. ATTR can be hereditary (ATTRh) or acquired as a wild type (ATTRwt) [1]. ATTR more commonly involves peripheral nerves and the heart, where deposition in the heart leads to thickened muscle, poor compliance with diastolic dysfunction, and conduction system disruption leading to arrhythmias [1,2].

Unfortunately, ATTR is a significantly underdiagnosed disease and is usually diagnosed late, but it should be suspected in anyone with heart failure with restrictive physiology [3]. ATTRwt mutations have been suggested to be more commonly associated with cardiomyopathy occurring in 70- to 80-year-olds while ATTRh occurs in 50- to 60-year-olds [3]. One study found that 25% of those over 80 years old with heart failure with preserved ejection fraction (EF) had ATTR deposits, and the likelihood of deposits increased with age [4].

The median survival of patients with ATTRwt and ATTRh is 31.6 and 34.1 months, respectively [2]. Another study stratified median survival from time of diagnosis by age and found that patients over 70 years old had a median survival of three years for both ATTRh and ATTRwt. For those diagnosed under 70 years old, median survival was 5.3 years for ATTRwt and 6.8 years for ATTRh [5].

Over 120 mutations of ATTR have been found with various phenotypic presentations [6]. Val30Met is the most common mutation, presenting primarily with neuropathy and rarely cardiomyopathy. It is associated with lower levels of brain natriuretic peptide (BNP), N-terminal pro-brain natriuretic peptide (NT-proBNP), and troponin T than non-Val30Met ATTRh [7-9]. Glu89Gln is the second most common mutation and is associated with lower EF, larger ventricular mass, and shorter E-wave deceleration time on echocardiogram compared to Val30Met ATTRh [8]. Of all the hereditary mutant variations, V122I has the greatest disease burden and morbidity in physical function, symptoms, social function, and quality of life, presenting primarily as advanced cardiomyopathy with preceding carpal tunnel syndrome in 4% of African Americans and Afro-Caribbean patients [2]. Due to the hereditary nature of these mutations, they tend to cluster in certain populations, and the majority are missense or nonsense mutations [9]. Higher disease severity is related to a higher ratio of ATTRh to ATTRwt, increasingly unstable ATTR production, and less ATTR degradation leading to higher levels of serum ATTR and more deposition in tissues [10].

There are two different staging systems for ATTR severity. The Mayo Clinic system defines ATTR according to stages I-III depending on whether none, one, or both troponin T and NT-proBNP are above the values of 0.05 ng/mL and 3000 pg/mL, respectively [11]. The four-year overall survival rate for stages I, II, and III were 57%, 42%, and 18%, respectively [11]. The European Society of Cardiology in 2017 used a similar I-III staging system with the same NT-proBNP requisite but replaced troponin T with an effective glomerular filtration rate cutoff of 45 mL/min. The median survival for the three stages was 69, 47, and 24 months [12].

## Case presentation

**Figure 1:**
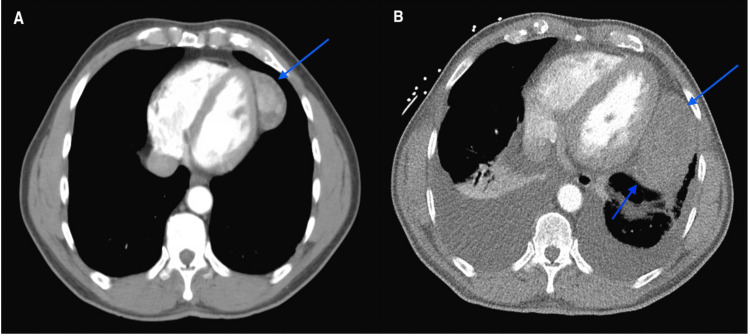
CT scan of the chest with IV contrast. (A) Mediastinal mass indicated by a blue arrow. (B) Nine-year interval mass growth delineated by blue arrows along with new pleural effusions. CT: computed tomography; IV: intravenous.

An African American male in his 70s with hypertension and hyperlipidemia who had undergone a workup for prostate adenocarcinoma was incidentally discovered to have a 5.1 x 3 cm left-sided mediastinal mass adjacent to the pericardium and lateral to the left ventricle (LV) on chest computed tomography (CT) (Figure 1A). There were no scrotal masses, muscular weakness, fevers, night sweats, or weight loss. Additional workup for the mass did not show positive emission tomography (PET) avidity, and laboratory tests were unremarkable. Although serial imaging showed progressive growth of the mass, he did not have any attributable symptoms and thus did not desire a biopsy.

Nine years after the incidental discovery, he presented to the hospital with increasing mild exertional dyspnea over several weeks where he was found to have a troponin of 0.06-0.75 ng/mL (reference <0.03 ng/mL), an electrocardiogram with right bundle branch block, and an increase in mass size to 8.4 x 4.6 x 7 cm with new pleural effusions on a repeat CT scan (Figure 1B). Thoracentesis improved his dyspnea, and fluid analysis was consistent with transudate effusion without malignant cells. A nuclear stress test showed global hypokinesis, EF of 35%, and biventricular hypertrophy, but no scar tissue or evidence of regional ischemia. The patient decided to forego catheterization because these findings were thought to be due to stress-induced cardiomyopathy and his symptoms had improved after thoracentesis.

**Figure 2:**
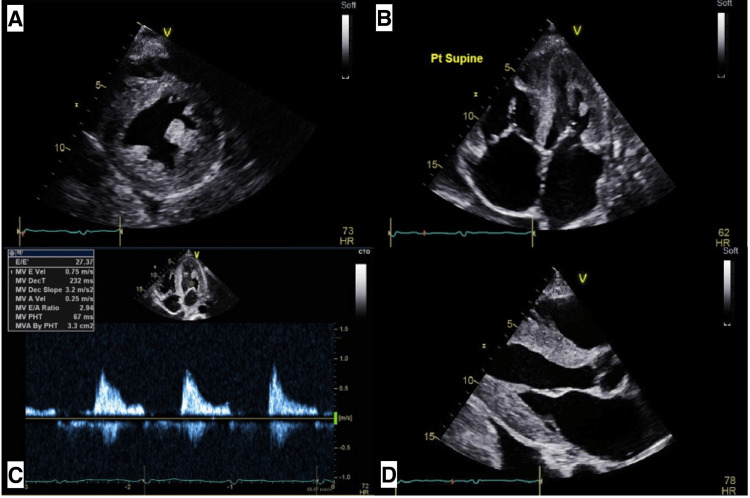
Transthoracic echocardiography. (A) Thickened LV on short axis view. (B) Bilateral atrial enlargement on apical view. (C) Grade 3 diastolic dysfunction. (D) LA dilation and LV hypertrophy on long axis view. LV: left ventricle; LA: Left atrial.

He was discharged to follow up with an outpatient transthoracic echocardiogram (TTE) (Figure 2) in tandem with cardiology consultation a month later, which showed a normal EF, moderate concentric hypertrophy, septal hypertrophy, moderate-severe mitral regurgitation, no communication of the heart with the mass, and grade 3 diastolic dysfunction, and thus, he was managed medically for nonischemic cardiomyopathy. Interval workup included a CT angiogram showing mass growth to 8.6 x 5.1 x 7 cm and hypervascularity suggestive of sarcoma with left internal mammary (LIMA), left inferior phrenic (LIPA), and questionably left circumflex arterial blood supply. After seeing a cardiothoracic surgeon, the patient again declined to pursue a biopsy or catheterization to evaluate arterial supply because his symptoms were not bothersome. He was thus managed conservatively with serial CT scans that continued to show slight interval progressions in mass size. 

About two years after his initial hospitalization, he presented to the emergency department for increasing lower extremity swelling and was found to have BNP 800 pg/mL (reference range <100 pg/mL) and troponin 0.10-0.14 ng/mL without electrocardiogram evidence of ischemia. A coronary steal by the mediastinal mass was more heavily deliberated; however, the patient felt better after heart failure optimization and declined catheterization.

**Figure 3:**
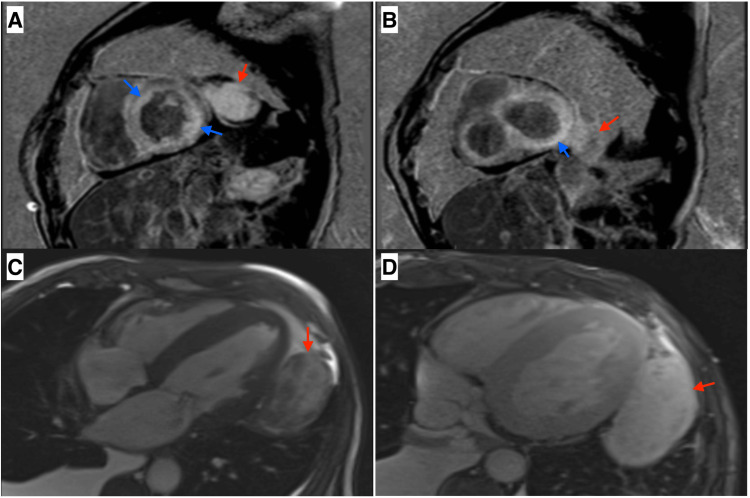
Cardiac MRI. Short axis view of the (A) basal ventricular and (B) mid-heart segments demonstrating with blue arrows global patchy diffuse mid-wall delayed enhancement, patchy infiltrate, and subendocardial distribution of the enhancement, typical of amyloidosis. Mediastinal mass delineated by red arrows showing (C) heterogeneity and (D) gadolinium-enhanced hyperintensity on T2.

He returned to the emergency department again a month later with a heart failure exacerbation with similar BNP, troponinemia without chest discomfort, and an electrocardiogram with new findings of a first-degree block, poor R-wave progression, and right bundle branch block. Elective catheterization demonstrated only mild-moderate coronary disease without intervenable lesions and the mass to be deriving its blood supply solely from the LIMA without coronary involvement. Since the patient symptomatically improved with diuretics, he was discharged to follow up with a cardiac MRI (CMRI) to ascertain heart muscle function and further evaluate chest wall involvement. This delineated the mass to be hyperintense, hypervascular, increased in size to 9.5 x 5.6 x 8.3 cm, abutting the lateral LV wall with a mild mass effect preventing full diastolic relaxation, and no chest wall involvement. The heart was also delineated to have EF 24%, restrictive physiology, and delayed gadolinium enhancement (Figure 3). Ultimately, outpatient management was difficult due to poor compliance with diet and medications.

**Figure 4:**
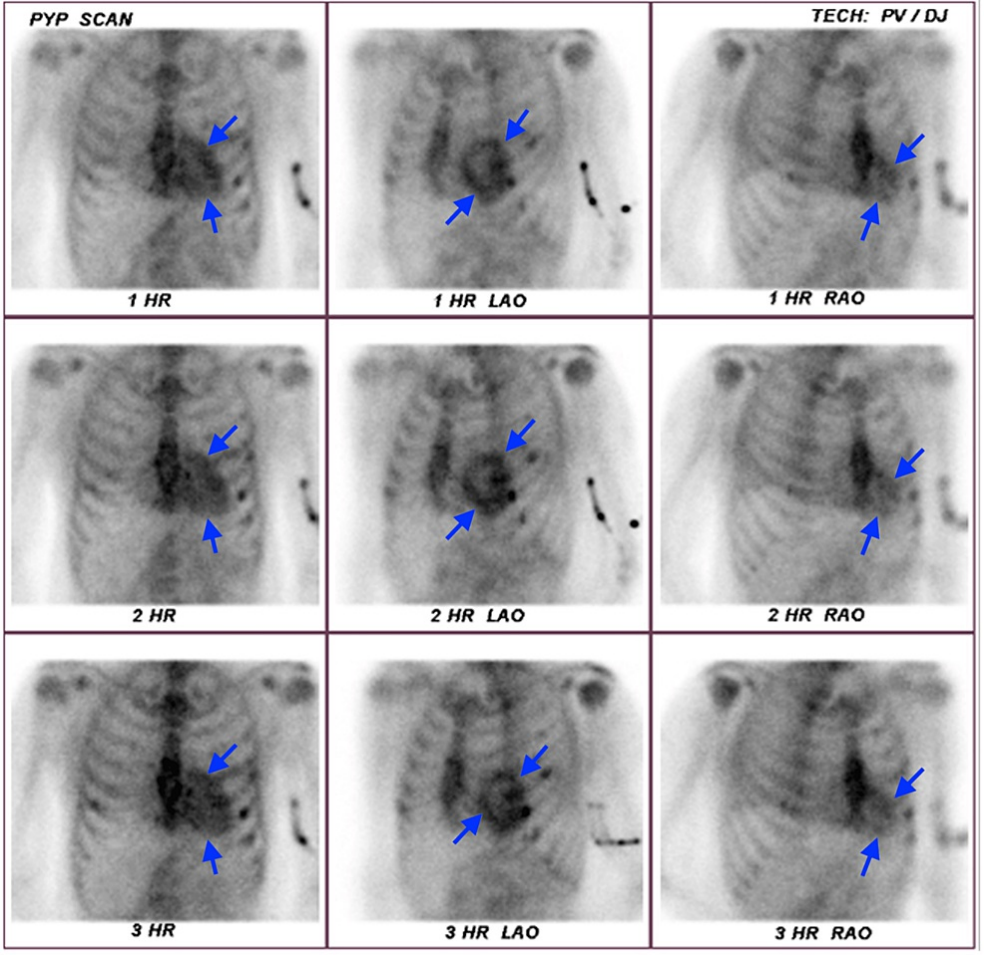
99mTc-PYP scintigraphy showing cardiac radiotracer uptake that is more pronounced than locoregional osseous uptake. 99mTc-PYP: technetium-99m pyrophosphate.

About a year after his catheterization, he suffered a stroke due to new atrial fibrillation. While attending outpatient speech therapy, he appeared to have briefly lost consciousness and was brought to an emergency department where evaluation revealed intermittent left lower chest discomfort, decompensated heart failure, atrial fibrillation, troponin 6 ng/mL without ST changes on electrocardiogram, and BNP 1800 pg/mL. After transfer to a catheterization center, he developed a brief cardiac arrest, and urgent catheterization again showed no intervenable lesions; however, the cardiac index was noted to be 1.5 L/m^2^. A right heart catheterization revealed moderate type 2 pulmonary hypertension and severe diastolic dysfunction. He was transferred to the ICU on dobutamine to continue management for decompensated heart failure. Follow-up TTE showed EF 30%-35%, bi-atrial enlargement, biventricular hypertrophy, ventricular paradoxical motion consistent with left bundle branch block, and severe right-sided pressures. The catheterization and echo findings in combination with the previous CMRI placed amyloidosis higher on the differential. Free light chains and serum and urine protein electrophoresis were unremarkable. Technetium-99m pyrophosphate (99mTc-PYP) scintigraphy showed increased cardiac uptake compared to the bone with a ratio of 1.55, consistent with amyloidosis (Figure 4). He was eventually discharged on chronic inotrope therapy but had several readmissions for decompensated heart failure and ultimately deteriorated to hospice care.

## Discussion

Diagnostic and management difficulties were encountered multiple times throughout this patient's care. It was unclear whether his symptomology was due to the mediastinal mass producing an effusion, compression, or involving the heart, resulting in heart failure. Attention was angled primarily on this mass being related to the heart failure readmissions, in addition to his poor compliance with diet and medications, clouding the consideration of other diagnoses such as amyloidosis. Amyloidosis was also not considered earlier on because this patient was largely asymptomatic until only two to three years before his death. The diagnosis of amyloidosis was entertained late, only after piecing together the results of catheterization, echo, and CMRI findings. These findings of restrictive physiology, diastolic dysfunction, later development of systolic dysfunction, delayed gadolinium enhancement, and absence of significant coronary artery disease prompted an amyloidosis workup.

It is difficult to elucidate whether a soft tissue mass is a slow-growing sarcoma or a benign tumor without tissue pathology. Anterior mediastinal soft tissue tumors may include over 50 histological subtypes, including vascular, lymphomatous, nervous, lipoid, muscular, and connective tissue derivatives [13]. Functional MRI favored the mass to be a sarcoma due to its hypervascularity, dynamic enhancement, large size, and heterogeneity, although there may be some overlap of these features between benign and malignant tumors [14,15]. Both low-grade sarcomas and benign tumors may demonstrate slow growth and lack fluorodeoxyglucose avidity on PET, as PET is primarily valuable in detecting aggressive tumors and malignant transformation [16]. Metastasis of his prostate adenocarcinoma to the mediastinum was low on the differential given the lack of metastasis anywhere else, especially in the bones. Altogether, the findings were more convincing of a low-grade sarcoma.

The prognosis of sarcomas is based on patient age, tumor size and depth, histological subtype, grade, and stage [17]. According to the Memorial Sloan Kettering Cancer Center postoperative nomogram, this patient would have scored 120 points at the time of diagnosis by having 60 years of age, a 5-10 cm tumor, and a deep thoracic location, without including histological scoring, indicating about a 10% chance of death from a low-grade sarcoma in 12 years [17]. Our patient's survival was beyond 15 years and likely would have continued if not for the cardiac amyloidosis.

Some cancers have a higher association with amyloidosis compared with others, stemming from a chronic inflammatory microenvironment [18,19]. Hematologic cancers involving plasma cell upregulation are associated with both AA and AL amyloidosis to a greater extent than solid tumors associated with AA amyloidosis [18,19]. Malignancies in general are often accompanied by a component of granulomatous inflammation that leads to AA amyloidosis [18,19]. Sarcoma is one of the possible causes of AA amyloidosis, but this patient's prostate cancer is not [19]. However, there was no mention of the frequency or degree of involvement between sarcoma and AA amyloidosis in this study or our literature search. Unfortunately, our center does not have a 99mTc or 123I labeled serum amyloid P component scintigraphy for AA amyloid detection so we cannot definitively say there was no AA amyloidosis secondary to the sarcoma.

Workup of ATTR-related cardiomyopathy includes echocardiography, which is neither sensitive nor specific. In advanced disease, it may show global muscular thickening, septal involvement, reduced EF, and diastolic dysfunction from restrictive cardiomyopathy [4]. According to the Mayo Clinic staging system, our patient was likely stage II by the time he presented to the hospital for the first time based on his elevated troponin of >0.1 ng/mL meeting the cutoff value of 0.05 ng/mL while the outpatient BNP never reached the threshold of 3000 pg/mL [12]. The stage may be falsely elevated if these values are only utilized during times of decompensated heart failure [12]. Speckle-tracking echocardiography may show a marked reduction in the longitudinal strain of the basal wall segments with apical sparing, which is highly sensitive and specific for cardiac amyloidosis [4]. CMRI with gadolinium will show enhancement of the entire subendocardial circumference and can diagnose ATTR amyloidosis with 80% sensitivity and 94% specificity [4]. This differentiates ATTR from hypertrophic cardiomyopathy, which does not have enhancement of the full endocardium [4]. The downsides of CMRI are the high degree of false positives and false negatives, the inability to be performed in all patients, and the inability to differentiate amyloidosis types [4]. Biopsy verification with immunostaining is essential to the diagnosis of ATTR, but it does not have to be a biopsy of the endomyocardium [1]. However, due to poor sensitivity (73%), negative biopsies do not rule out ATTR, especially ATTRwt [1]. The gold standard for diagnosis is mass spectroscopy showing the exact amyloid present and genotyping the exact variation; however, this may miss unknown mutations and variations that are not in conventional databases [1].

A more recent development is radionuclide scintigraphy with either 99mTc-diphosphono-propanodicarboxylic acid, 99mTc-pyrophosphate, or 99mTc-hydroxymethylene-diphosphate, which have 97% sensitivity and 100% specificity for ATTR amyloidosis [4]. They may also detect disease before CMRI and echocardiogram as well as characterize disease severity with tracer retention [4]. Scintigraphy can also be useful to differentiate ATTR from AL or cardiac apolipoprotein A-I amyloidosis due to their low-grade uptake [4]. Although we do not have tissue for mass spectroscopy or genotyping to prove our patient had ATTR, it is the likely culprit based on 99mTc-PYP uptake, cardiomyopathy phenotype without extracardiac involvement, negative protein electrophoresis, and free light chain workup, and no other underlying inflammatory conditions, plasma cell dyscrasias, or chronic renal failure.

Drugs to treat ATTR are limited, but this is a very active field of research. Diflunisal is a nonsteroidal anti-inflammatory drug that was found to inhibit the progression of peripheral neuropathy and cardiomyopathy, with a risk of thrombocytopenia and a decline in renal function [1]. Tafamidis is a more recent drug that functions by binding to transthyretin, inhibiting the dissociation of its quaternary structure to form amyloid fibrils [1,20]. It is associated with lower all-cause mortality, cardiovascular-related hospitalizations, and a lower rate of decline in distance for a six-minute walk test [20]. The benefits of tafamidis have been found in earlier disease stages, emphasizing the importance of early ATTR diagnosis [20]. Current research is also investigating drugs that inhibit ATTR production in the liver, such as siRNA that silences gene expression and antisense oligonucleotide drugs that bind mRNA to mark for destruction [1]. Patisiran is a double-stranded synthetic oligonucleotide that improves neuropathy scores, 10-minute walk test results, NT-proBNP, and longitudinal LV strain levels and was approved in 2018 for ATTRh-associated peripheral neuropathy [20]. Inotersen is a single-stranded antisense oligonucleotide inhibitor of ATTRh and ATTRwt approved for polyneuropathy treatment in Europe; however, it is associated with an increased risk of glomerulonephritis and thrombocytopenia [20]. Doxycycline has also been found to disrupt fibrils and reverse aggregated amyloid [1]. 

Earlier attempts at treating ATTR amyloidosis were focused on replacing the source of ATTR with normal TTR by liver transplant, with the hope that this would cure the disease [1]. However, studies have shown that deposition continues post-liver transplant with ATTRwt, and those undergoing both liver and heart transplants have better survival outcomes than those with liver transplants alone [1]. This may be due to ATTRh deposits forming a nidus for new ATTRwt [1]. One may also consider an isolated heart transplant if the patient has the V122I variant, which often does not include deposition of ATTR outside of the heart [1].

## Conclusions

Amyloidosis is a spectrum of diseases related to either familial genetics, acquired mutations, or long-standing diseases. Diagnosis requires a scrutinous eye, especially in the face of other pathologies that may seem to confound a patient’s presentation, as in our patient with progressive restrictive diastolic heart failure who also had a sarcomatous mass adjacent to the LV. Even though the gold standard has been tissue biopsy examination, noninvasive imaging modalities such as echocardiogram, CMRI, and 99mTc scintigraphy yield high specificity. Amyloidosis may be a rare entity, but all restrictive cardiomyopathies must include workup for this pathology, so newly evolving treatments may be offered early.
